# Supplementary material for: Sleep Health, Individual Characteristics, Lifestyle Factors, and Marathon Completion Time in Marathon Runners: A Retrospective Investigation of the 2016 London Marathon
Source: Brain Sci. 2023 Sep 20;13(9):1346. doi: 10.3390/brainsci13091346 (PMC10527296; doi:10.3390/brainsci13091346)
Supplement: Supplementary file 1 [file brainsci-13-01346-s001.zip › brainsci-2545256-supplementary.pdf]

Supplementary Table S1. Interactive Influence of Age and Sex on Sleep Characteristics

IV	β	SE	*p* value	$_p\eta^2$	95% CI [LL, UL]
**Sleep Difficulty Score**					
Age	0.35	0.20	0.11	0.005	[-0.08, 0.78]
Sex	0.00	0.20	1.00	0.000	[-0.42, 0.42]
Age x Sex	0.09	0.40	0.81	0.000	[-0.68, 0.86]
**Total Sleep Time**					
Age	0.12	0.10	0.09	0.009	[-0.02, 0.26]
Sex	-0.20	0.10	0.002	0.009	[-0.35, -0.08]
Age x Sex	0.16	0.10	0.20	0.002	[-0.09, 0.41]
**Satisfaction**					
Age	0.15	0.10	0.08	0.010	[-0.02, 0.32]
Sex	0.00	0.10	0.89	0.001	[-0.18, 0.15]
Age x Sex	0.22	0.20	0.16	0.002	[-0.09, 0.52]
**Sleep Onset Latency**					
Age	-0.20	0.10	0.009	0.017	[-0.3, -0.04]
Sex	0.01	0.10	0.90	0.001	[-0.12, 0.13]
Age x Sex	-0.20	0.10	0.17	0.002	[-0.39, 0.07]
**Maintenance**					
Age	0.21	0.10	0.003	0.011	[0.07, 0.35]
Sex	0.15	0.10	0.03	0.005	[0.01, 0.29]
Age x Sex	-0.10	0.10	0.57	0.000	[-0.33, 0.18]
**Medication Use**					
Age	0.04	0.00	0.16	0.001	[-0.02, 0.09]
Sex	0.07	0.00	0.01	0.006	[0.02, 0.12]
Age x Sex	-0.10	0.1	0.28	0.001	[-0.15, 0.04]

Supplementary Table S1 presents the results from analyses that explored an interactive relationship between age and sex on sleep characteristics. Age group (young vs. middle-aged adults), sex (male vs. female), and an interaction term between the two variables were included in each regression. Sleep characteristics derived from the Athlete Sleep Screening Questionnaire include the sleep difficulty score (SDS) and the items that comprise the SDS: Total sleep time (TST), sleep satisfaction (Satisfaction), sleep onset latency (SOL), sleep maintenance issues (Maintenance), and sleep medication use (Medication). Higher scores are reflective of more severe sleep problems. Means (standard deviation) are provided for each group across all sleep characteristics. Regression coefficient (β), standard error (SE), *p* value, partial eta squared ($_p\eta^2$), and the lower limit (LL) and upper limit (UL) from the 95% confidence interval (CI) are provided from each regression.
